# The Human Microbiome in Intensive Care - A Journey Forward?

**DOI:** 10.2478/jccm-2023-0032

**Published:** 2023-11-14

**Authors:** Leonard Azamfirei

**Affiliations:** Department of Anesthesia and Intensive Care, George Emil Palade University of Medicine, Pharmacy, Science and Technology, Târgu Mureș, Romania

The human microbiome, defined as a personal, genomic signature of our latent or manifest infectious profile (bacterial, viral, fungal), located predominantly in the digestive tract, opens the door to personalized medicine studies on a scale larger than the human genome in terms of data that can be analyzed and interpreted. Compared to the human genome, which has approximately 23,000 genes, the European Metagenomics of the Human Intestinal Tract and the Human Microbiome Project have reported 3.3 million non-redundant microbial genes [1].

The vast majority of normal gut microbiota is composed of four major phyla: Firmicutes (Gram-positive aerobic cocci: *Enterococcus* spp., and bacilli: *Clostridium* spp., 60–75%), Bacteroidetes (anaerobic Gram-negative bacteria: *Bacteroides* spp. and *Prevotella* spp., 30–40%), Actinobacteria (anaerobic Gram-positive bacteria: *Bifidobacterium*), and Proteobacteria (aerobic and anaerobic Gram-negative enterobacteria), as well as fungal mycobiota (*Candida* spp., *Saccharomyces* spp., *Candida albicans*) and viral microbiota [2]. Dysbiosis is the consequence of an imbalance between commensal microbes (Firmicutes, Bacteroidetes), which decline, and pathogenic microbes (Proteobacteria), which proliferate.

Enterotypes can be distinguished by specific biomarkers; however, due to the large differences in intestinal enterobiota between healthy individuals and ICU patients, these must be characterized separately. In this context, two microbiota patterns have been proposed for ICU settings: one that includes *Bacteroides* and certain *Enterobacteriaceae* correlated with high serum lactate levels (specific to septic patients), and another predominantly including *Enterococcus* [3].

The dynamics of the microbiome - its dependence on various normal or pathological factors, and particularly its behavior in relation to certain diseases—make the microbiome an important “organ” in intensive care, albeit still insufficiently studied. On the other hand, manipulation of the microbiota to reverse dysbiosis through targeted antibiotic therapy capable of eliminating selected microbiota, as well as the use of probiotics and prebiotics to restore balance in favor of beneficial bacteria, has inspired numerous studies assessing their effectiveness [4].

The factors affecting microbiota changes in ICU patients relate not only to severe organ pathology or multiple organ dysfunction but also to antibiotic usage, and intestinal transit disorders including enteral feeding. Antibiotic-induced dysbiosis is well documented for various drug classes such as lincosamides, fluoroquinolones, macrolides, and glycopeptides; it can also occur following the use of NSAIDs, proton pump inhibitors, vasopressors, or opioids [5].

The resistance to colonization of an intact microbiota enhances the ability to combat multidrug-resistant (MDR) bacteria and is directly related to immune status, particularly innate immunity, and less so to adaptive immunity. Dysbiosis alters the permeability of the intestinal mucosa leading to increased systemic passage of bacterial components, metabolites, and pathogen-associated molecular patterns (PAMPs) which in turn triggers the release of pro-inflammatory mediators [6].

The remote impact of intestinal dysbiosis on ICU patients is demonstrated in specific pathologies. For instance, intestinal microbiota appears to influence tracheal microbiota in mechanically ventilated patients, with a higher incidence of *Bacteroides* spp., an anaerobic gut bacterium, found in the lungs of patients with ventilator-associated pneumonia (VAP) [7], raising the hypothesis of a bidirectional axis between the gut and the lung [8]. Although the link between intestinal dysbiosis and nosocomial infections has been hypothesized, it has not been conclusively confirmed [9]. We are at the present stage where we can consider that alterations in microbiota in critically ill patients make them more susceptible to immune dysfunction and we can identify a decrease in bacterial diversity in critically ill patients, especially in septic patients [10].

Systemic decontamination of the digestive tract (SDD), typically performed through a combination of local administration of non-absorbable antibiotics and a short course of systemic cephalosporin therapy, aims to prevent colonization by pathogenic microorganisms while preserving anaerobic bacteria. The positive effect of this approach, already used widely, has led to a lower rate of infections and mortality rates but has also had the negative effect of increasing bacterial resistance [10].

Methods to prevent intestinal dysbiosis, such as the use of beta-lactamase enzymes and non-specific adsorbent activated charcoal, are in varying phases of study linked to specific classes of antibiotics. Probiotics (e.g., *Saccharomyces boulardii*, *Lactobacillus* spp., *Bifidobacterium* spp.) have not proven to be as effective as expected. While their use has been associated with a decrease in the incidence of VAP, survival rates have not significantly improved [11]. In addition to these methods, microbiota transplantation has been used as a therapy, especially for *Clostridium difficile* infections.

Few studies address microbiota in ICU patients, and even fewer link microbiota to personalized medicine, whose importance in the ICU may be key to the future treatment of critically ill patients [12, 13]. The link may be mediated through inflammatory markers (oxygen and nitrogen reactive species, cytokines with genetic polymorphisms that modulate immune response in sepsis and chemokines), metabolites (especially microbial branched-chain amino acids), and gene toxicity [14, 15].

The answers may be found through deep sequencing techniques that identify major differences between individuals, as well as longitudinal studies that capture the dynamics of these microbiotic profiles. The potential for microbiota to serve as biomarkers will be confirmed only by leveraging machine learning capabilities for data processing [16,17,18].

Whether the microbiota will become a next-generation therapeutic tool depends on our ability to characterize the microbiome and integrate it into the advancing genomic revolution that is currently unfolding.
